# The Impact of Raising Children with Barth Syndrome on Parental Health-Related Quality of Life and Family Functioning: Preliminary Reliability and Validity of the PedsQL™ Family Impact Module

**DOI:** 10.1155/2023/5588935

**Published:** 2023-12-30

**Authors:** Yoonjeong Lim, Ickpyo Hong, Areum Han

**Affiliations:** ^1^Division of Occupational Therapy, Binghamton University, Johnson City, NY 13790, USA; ^2^Department of Occupational Therapy, Yonsei University, Wonju 26493, Republic of Korea; ^3^Department of Occupational Therapy, University of Alabama at Birmingham, Birmingham, AL 35294, USA

## Abstract

**Objective:**

This study examined the preliminary reliability and validity of the PedsQL™ Family Impact Module (PedsQL FIM) in families of children with Barth syndrome (BTHS).

**Method:**

A total of 72 parents with children or youth between the ages of 5 and 19 participated in this study. Thirty-three parents of children with BTHS and 39 parents of unaffected children completed the PedsQL FIM and a demographic information form. Internal consistency reliability and item-total correlations were calculated to test the reliability of the PedsQL FIM. Construct validity was examined using the known-groups method. We estimated the mean score differences of the PedsQL FIM between the two groups using three different models, including unadjusted, multivariate regression, and propensity score matching with inverse probability of treatment weighting (PS-IPTW) models.

**Results:**

The Cronbach's alpha coefficients were greater than 0.70 for all scales of the PedsQL FIM, except for the communication scale. The item-total correlations were significant for all scales with moderate to high correlations (*p* < .05). In construct validity, the mean scores of the PedsQL FIM between the two groups were significantly different (*p* < .05) for all scales and total score in the unadjusted and PS-IPTW models. However, in the multivariate regression model, the family relationships scale was not significant between the two groups.

**Conclusion:**

The PedsQL FIM demonstrated adequate measurement properties of preliminary reliability and validity in assessing the impact of children with BTHS on parental health-related quality of life (HRQoL) and family functioning. Further research needs to be conducted to examine the psychometric properties of the PedsQL FIM with a large sample of BTHS and with other pediatric rare diseases.

## 1. Introduction

With the rapid advancement of knowledge in the field of human genetics, it has become increasingly imperative for occupational therapists to possess a comprehensive understanding of rare genetic disorders to effectively provide healthcare services to their clients [1]. Barth syndrome (BTHS) is a rare, X-linked disorder found primarily in boys [2]. In the United States, BTHS occurs in approximately 1 in every 1,000,000 births [3]. BTHS is characterized by cardiomyopathy, neutropenia, muscle weakness, growth delays, and exercise intolerance [4, 5].

Cardiomyopathy typically manifests during the first two years, predominantly affecting males who exhibit left ventricular dysfunction and have symptoms indicative of heart failure [6, 7]. Neutropenia refers to reduced neutrophils in white blood cells, which play an important role in guarding against bacterial infections [8, 9]. BTHS displays skeletal muscle weakness and easy onset of fatigue; participating in physical activities is difficult and requires great energy [4, 10]. Gross motor development in BTHS is delayed, and children with BTHS typically have small stature and low weight in their childhood, both of which are below average [11]. BTHS is classified as a mitochondrial disease exhibiting muscular and neurological problems, leading to mitochondrial dysfunction and deficient cardiolipin [8, 11]. Mitochondria is a main producer of energy in cells, and cardiolipin deficiency leads to mitochondria not generating enough essential lipids crucial for maintaining mitochondrial structure leading to muscle weakness and exercise intolerance [12, 13]. There is no cure or specific treatment yet available for BTHS. Therefore, their families need life-long support to cope with the daily caregiving challenges they might face.

Storch et al. [7] reported that parents of children with BTHS have significantly higher stress and distress levels than parents of unaffected children. In addition, Lim [14] reported that parental health-related quality of life (HRQoL) and family functioning of the parents of children with BTHS were significantly lower than those of unaffected children. HRQoL is a multidimensional construct encompassing physical, emotional, cognitive, and social functioning that impacts an individual's overall health and wellness [15, 16]. Quality of life, which is often interchangeably used with HRQoL for certain constructs, is one of the important target outcomes of occupational therapy to enhance clients' ability to engage in desired occupations as well as their caregivers [17]. Several studies found that parental HRQoL and family functioning are affected by their child's health conditions [14, 18–20]. The measurement of parental HRQoL could provide significant insights into addressing the healthcare needs of families as they confront the difficulties associated with their child's illness. However, identifying the relationship among a child's illness, the course of the illness, and the impact on the family is a multifaceted and intricate process [21]. Therefore, choosing an optimal instrument to assess the impact of the child's health condition on families, including parents in particular, is critically important.

The PedsQL FIM assesses parental HRQoL and family functioning [22], which has been reported as a reliable and valid measure in assessing the impact of a child's health condition on parental HRQoL and family functioning [21–25]. In rare disease populations, however, there is a lack of evidence of the psychometric properties of the PedsQL FIM. According to the current Standards for Educational and Psychological Testing, it is imperative to present evidence of the reliability and validity of an instrument when utilized for a particular population [26]. Therefore, this study examined the preliminary reliability and validity of the PedsQL FIM in families of children with BTHS. We hypothesized that the PedsQL FIM would distinguish between families of children with BTHS and those with unaffected children.

## 2. Methods

The PedsQL FIM data were previously analyzed and reported to investigate the impact of raising children with rare diseases on parental quality of life and family functioning [14]. However, the reliability and validity of the instrument were assumed and not fully reported in the previous study. In this study, the item and scale scores were reanalyzed to examine the preliminary reliability and validity of the PedsQL FIM in families of children with BTHS.

### 2.1. Participants

A total of 72 parents with children or youth between the ages of 5 and 19 participated in this study. Inclusion criteria included (1) parents of children with BTHS, or unaffected children, and (2) parents who were English-speaking and able to read at an 8th-grade level. Thirty-three parents of children with BTHS were recruited by the Barth Syndrome Foundation. Thirty-nine parents of unaffected children were recruited by word of mouth and by flyers posted on the University of Florida (UF) department websites and Listserv, Facebook groups, and HealthStreet. This study was approved by the UF Institutional Review Board. Written informed consent was obtained from each parent.

### 2.2. Instrument

#### 2.2.1. The PedsQL™ Family Impact Module

The PedsQL™ Family Impact Module (PedsQL FIM) is a 36-item questionnaire assessing the impact of pediatric chronic health conditions on parental HRQoL and family functioning [22]. This assessment consists of 8 scales: (1) physical functioning, (2) emotional functioning, (3) social functioning, (4) cognitive functioning, (5) communication, (6) worry, (7) daily activities, and (8) family relationships. Each item is scored on a 5-point scale (never a problem = 0, almost never = 1, sometimes = 2, often = 3, and almost always = 4) and transformed to a 0 to 100 scale (never a problem = 100, almost never = 75, sometimes = 50, often = 25, and almost always = 0). The total score of the PedsQL FIM is calculated using the mean of the 36 items. Within the PedsQL FIM, the Parental HRQoL score is calculated by the mean of the items in the physical functioning, emotional functioning, social functioning, and cognitive functioning. The family functioning score is calculated by the mean of the items in daily activities and family relationships. Higher scores indicate better parental HRQoL and family functioning.

#### 2.2.2. The Demographic Information Form

The demographic information form, generated by the investigators of this study, consisted of (1) the child's date of birth and age, (2) marital status, (3) race, (4) employment, (5) level of education, and (6) family income.

### 2.3. Statistical Analysis

The child and parent demographic variables and study variables were analyzed with an independent-samples *t*-test for continuous variables and with Chi-square or Fisher's exact test for categorical variables. Internal consistency reliability was calculated with Cronbach's alpha. Scales with coefficient values greater than 0.70 were considered acceptable group-level analyses [27]. Additionally, item-total correlations were calculated using the Pearson correlation coefficient to assess the relationship between each item comprising scales and the scale's total score.

Construct validity was determined using the known-groups method [28]. The scale scores of the PedsQL FIM were compared between the parents of children with BTHS and parents of unaffected children. In the analysis, we estimated the mean score differences between the two groups using three different models including an unadjusted model (independent-samples *t*-test), multivariate regression model, and propensity score matching with inverse probability of treatment weighting (PS-IPTW) [29, 30]. Since the two groups would likely have heterogeneous demographic characteristics and those confounding factors could lead to biased point estimates [29, 30], the PS-IPTW method was applied to control for the demographic differences between the two-comparison groups. When the demographic characteristics were not balanced after the PS-IPTW, those variables were additionally controlled for the group comparisons [31]. Statistical analyses were conducted using SAS statistical software version 9.4 (SAS Institute, Inc.). The alpha level was set at.05.

## 3. Results

Among 72 parents, 33 parents of children with BTHS and 39 parents of unaffected children completed the PedsQL FIM and demographic information form. There were two missing data in the parent employment status, one person each from the groups.

### 3.1. Demographic Characteristics

Table 1 displays summary statistics for child and parent demographic variables. Four out of 7 demographic variables were significantly different between the two groups, including child sex, parent race, education, and employment status (all *p* < .05). Since BTHS is an X-linked disorder that occurs in the male population, the BTHS group in this study consisted of males only, which caused the child sex variable significantly different between the two groups. In the parent demographic variables, 20 (51.30%) parents were Non-Hispanic White in the unaffected group versus 31 (93.94%) in the BTHS group. Similarly, 31 (79.49%) of parents in the unaffected group had a graduate or professional degree, as compared to 11 (33.33%) in the BTHS group. Thirty-one of the parents (79.49%) in the unaffected group were employed full-time versus 18 parents (54.55%) of the children with BTHS. Based on these results, construct validity was examined by controlling for those significant demographic variables between the two groups using the PS-IPTW methods in the following section.

### 3.2. Reliability

Table 2 presents the internal consistency reliability and item-total correlations of the PedsQL FIM for the unaffected and BTHS groups. The PedsQL FIM demonstrated strong internal consistency. The Cronbach's alpha coefficients were greater than 0.70 for all scales, except for the communication in the BTHS group. The item-total correlations were all significant with moderate to high correlations for both groups (all *p* < .05).

### 3.3. Construct Validity

Before applying the PS-IPTW method, child sex, parent race, education, and employment status were significantly different between the two groups (all *p* < .05). After applying the PS-IPTW method, all demographic characteristics were balanced (all *p* > .05), except for parent race (*p* = .0216) and parent education (*p* = .0033). These unbalanced characteristics were adjusted again when comparing the group differences.  Table 3 displays the mean score differences of the PedsQL FIM between the BTHS and unaffected groups using unadjusted, multivariate regression, and PS-IPTW adjustment models. Regardless of the estimation method, the mean scores between the two groups were significantly different in the unadjusted and PS-IPTW models (*p* < .05), except for the family relationships in the multivariate regression model.

## 4. Discussion

This study investigated the preliminary reliability and validity of the PedsQL FIM in measuring parental HRQoL and family functioning of children with BTHS. The PedsQL FIM demonstrated adequate measurement properties of preliminary reliability and validity in assessing the impact of children with BTHS on parental HRQoL and family functioning. The significant differences in the demographic characteristics between the two groups (e.g., employment status) were controlled for by applying the PS-IPTW method, which improved the methodological rigor of the analysis.

In reliability, Cronbach's alpha coefficients in both groups exceeded the minimum expected value for group-level analyses of 0.70 [27], except for the communication scale in the BTHS group. Likewise, previous psychometric studies for the PedsQL FIM demonstrated the lowest Cronbach's alpha value in the communication scale [21, 22, 24, 25, 32]. The item-total correlations were significant in both groups (all *p* < .05), indicating that each item that makes up scales is correlated with appropriate scales with moderate to high relationships [23]. Although the communication scale of the BTHS group demonstrated Cronbach's alpha coefficient of 0.56, the item-total correlations ranged from .637 to .722 (*p* < .01) with high correlation, meaning that the three items comprising the communication scale were significantly correlated with the scale. Scarpelli et al. [25] investigated the psychometric property of the PedsQL FIM for families with pediatric cancer and found that Cronbach's alpha of a few scales including the communication was below 0.70. They suggested that these scales should be exclusively utilized only for descriptive or exploratory studies. While the communication scale did not meet the minimum standard value for group-level analyses, the findings still provide supporting evidence for the preliminary reliability of the PedsQL FIM for families of children with BTHS, which is consistent with the previous studies that examined the reliability of the instrument for pediatric chronic health conditions [21, 22, 24].

In construct validity, the mean scores of the PedsQL FIM between the parents of children with BTHS and those of unaffected children were significantly different for all scales (*p* < .05) both in the unadjusted and PS-ITPW models. In other words, the scores of the parental HRQoL and family functioning in the BTHS group were significantly lower than those in the unaffected group, indicating that the presence of their child's disease entails constraints and challenges in the overall functioning of the entire family unit [22]. In the multivariate regression model, however, the family relationships scale was nonsignificant between the groups (*p* = .1163). In the unadjusted model using independent-samples *t*-test, the standard error of the mean difference of the family relationships scale between the two groups was 4.92 whereas the standard error was increased to 6.28 in the multivariate regression model. In other words, while the mean differences between the unadjusted and multivariate regression models were fairly consistent, there was a 27.6% increase in the standard error in the multivariate regression model, leading to the nonsignificant estimation of the family relationships scale. Typically, standard error could be inflated by a small sample size and multicollinearity among study variables [33]. Since this study utilized a relatively small sample size of 72 for the multivariate regression model, further research with a larger sample size would be needed to examine if the family relationships scale distinguishes between the BTHS and unaffected groups. Although the family relationships scale was not significantly different between the two groups after controlling for demographic variables in the multivariate regression model, the finding still provides appropriate evidence for the construct validity of the PedsQL FIM in the BTHS group, which is consistent with a previous study that compared mean scores of the PedsQL FIM between parents of children with and without chronic health conditions [21].

There are several limitations to this study. Due to the generic issue of rare disease research (e.g., low prevalence), this study is limited by the relatively small sample size. Since this study used convenient sampling, selection bias might occur in choosing participants. This cross-sectional study collected the data at a one-time point, so we could not examine the stability of the information using the test-retest reliability. The participants of this study were mostly white, middle-class, and highly educated. By applying the PS-IPTW method, however, the heterogeneous demographic characteristics of the participants were controlled for the analysis. Future studies should conduct the psychometric property of the PedsQL FIM with a large BTHS sample and other pediatric rare diseases. Additionally, the measurement of criterion and convergent validity of the PedsQL FIM would provide the degrees to which the instrument correlates with other established family impact instruments and pediatric rare disease symptom assessments.

## 5. Conclusions

This study provided evidence of good preliminary reliability and validity of the PedsQL FIM in measuring the impact of children with BTHS on parental HRQoL and family functioning. By applying the PS-IPTW method, this study enhanced the methodological rigor in examining the reliability and validity of the PedsQL FIM. In addition, measuring family impact using the PedsQL FIM will help better understand the impact of children with BTHS on parental HRQoL and family functioning. It will also provide crucial information for health professionals and researchers to develop holistic and comprehensive healthcare services for families.

## Figures and Tables

**Table 1:** Summary statistics for child and parent demographic variables.

Characteristics	Total (*N* = 72)	Unaffected (*n* = 39)	BTHS (*n* = 33)	*p* value
Child age	11.10 (4.12)	10.74 (3.64)	11.53 (4.65)	.4245
Child sex				.0005
Male	60 (83.33)	27 (69.23)	33 (100.00)	
Female	12 (16.67)	12 (30.77)	0 (0.00)	
Parent race				<.0001
Non-Hispanic White	51 (70.83)	20 (51.28)	31 (93.94)	
Other	21 (29.17)	19 (48.72)	2 (6.06)	
Parent education				<.0001
Graduate or professional degree	42 (58.33)	31 (79.49)	11 (33.33)	
Less than graduate or professional degree	30 (41.67)	8 (20.51)	22 (66.67)	
Marital status				.4935
Married	61 (84.72)	32 (82.05)	29 (87.88)	
Other	11 (15.28)	7 (17.95)	4 (12.12)	
Employment status				.0212
Full-time	49 (68.06)	31 (79.49)	18 (54.55)	
Less than full-time	21 (29.17)	7 (17.95)	14 (42.42)	
Missing	2 (2.78)	1 (2.56)	1 (3.03)	
Family income				.6502
$80,000 or above	37 (51.39)	21 (53.85)	18 (46.15)	
Less than $80,000	35 (48.61)	16 (48.48)	17 (51.52)	

Note: *M* (SD) for child age; *n* (%) for the others (e.g., parent race). Categorical variables were analyzed by the Chi-square test or Fisher's exact test. Continuous variables were analyzed by independent *t*-test.

**Table 2:** Internal consistency of reliability and item-total correlations of the PedsQL FIM.

Scale (number of items)	Unaffected (*n* = 39)		BTHS (*n* = 33)	
	Cronbach's *α*	*r*_it_	Cronbach's *α*	*r*_it_
Parental HRQoL (20)	0.94	.477^∗∗^-.846^∗∗^	0.96	.585^∗∗^-.874^∗∗^
Physical functioning (6)	0.87	.713^∗∗^-.853^∗∗^	0.93	.761^∗∗^-.954^∗∗^
Emotional functioning (5)	0.88	.716^∗∗^-. 901^∗∗^	0.90	.793^∗∗^-.910^∗∗^
Social functioning (4)	0.88	.848^∗∗^-.884^∗∗^	0.92	.866^∗∗^-.916^∗∗^
Cognitive functioning (5)	0.91	.798^∗∗^-.907^∗∗^	0.96	.902^∗∗^-.962^∗∗^
Communication (3)	0.78	.810^∗∗^-.888^∗∗^	0.56	.637^∗∗^-.722^∗∗^
Worry (5)	0.92	.794^∗∗^-.940^∗∗^	0.80	.667^∗∗^-.830^∗∗^
Family functioning (8)	0.94	.727^∗∗^-.913^∗∗^	0.88	.578^∗∗^-.832^∗∗^
Daily activities (3)	0.81	.807^∗∗^-.913^∗∗^	0.94	.904^∗∗^-.967^∗∗^
Family relationships (5)	0.95	.879^∗∗^-.938^∗∗^	0.93	.809^∗∗^-.936^∗∗^
Total (36)	0.96	.434^∗∗^-.814^∗∗^	0.96	.349^∗^-.836^∗∗^

Note: *r*_it_ = item-total correlations. ^∗^*p* < .05. ^∗∗^*p* < .01.

**Table 3:** Mean score differences of the PedsQL FIM between the unaffected and BTHS groups.

	Unadjusted^a^				Multivariate adjustment^b^		PS-IPTW adjustment^c^	
	Unaffected (*n* = 39)	BTHS (*n* = 33)	Mean difference (SE)	*p*	Mean difference (SE)	*p*	Mean difference (SE)	*p*
Parental HRQoL	76.67 (15.04)	61.40 (21.59)	15.27 (4.46)	.0012	16.27 (5.42)	.0039	25.65 (6.90)	.0005
Physical functioning	74.78 (16.66)	60.60 (26.29)	14.18 (5.29)	.0099	13.01 (6.39)	.0461	21.73 (9.16)	.0214
Emotional functioning	75.64 (16.66)	59.39 (21.49)	16.24 (4.50)	.0006	18.53 (5.76)	.0021	27.51 (6.16)	<.0001
Social functioning	78.20 (21.64)	61.09 (27.12)	17.10 (5.74)	.0040	19.97 (7.10)	.0066	34.91 (8.67)	.0002
Cognitive functioning	78.07 (16.04)	63.78 (25.28)	14.28 (5.09)	.0071	13.24 (6.43)	.0437	18.90 (8.59)	.0322
Communication	87.39 (15.51)	59.09 (17.25)	28.30 (3.86)	<.0001	30.13 (4.90)	<.0001	38.01 (5.20)	<.0001
Worry	85.25 (19.89)	47.72 (19.32)	37.52 (4.64)	<.0001	41.14 (5.93)	<.0001	48.32 (6.26)	<.0001
Family functioning	70.96 (19.68)	57.23 (21.45)	13.73 (4.85)	.0061	14.56 (6.16)	.0213	30.63 (6.74)	<.0001
Daily activities	69.23 (21.04)	49.74 (19.32)	19.48 (6.44)	.0038	21.21 (7.91)	.0094	41.38 (10.50)	.0002
Family relationships	72.69 (20.89)	62.53 (20.72)	10.15 (4.92)	.0429	10.01 (6.28)	.1163	21.75 (6.73)	.0021
Total score	77.65 (14.79)	58.36 (18.03)	19.29 (3.86)	<.0001	20.71 (4.86)	<.0001	31.26 (5.59)	<.0001

Note: *M* (SD) for unaffected and BTHS; propensity score matching with inverse probability of treatment weighting (PS-IPTW); standard error (SE). ^a^Independent-samples *t*-test between the unaffected and BTHS groups. ^b^Multiple regression model controlling for the demographic characteristics listed in Table 1. ^c^After applying propensity score matching with inverse probability of treatment weighting, all demographics were balanced between the unaffected and BTHS groups.

## Data Availability

The data used for this study are available from Yoonjeong Lim, the first author of this manuscript. Please contact her at ylim3@binghamton.edu to request the data.

## References

[1] Schroeder-Smith K., Tischenkel C., Delange L., Lou J. Q. (2002). Duchenne muscular dystrophy in females: a rare genetic disorder and occupational therapy perspectives. *Occupational Therapy in Health Care*.

[2] Barth P. G., Scholte H. R., Berden J. A. (1983). An X-linked mitochondrial disease affecting cardiac muscle, skeletal muscle and neutrophil leucocytes. *Journal of the Neurological Sciences*.

[3] Miller P. C., Ren M., Schlame M., Toth M. J., Phoon C. K. L. (2020). A Bayesian analysis to determine the prevalence of Barth syndrome in the pediatric population. *The Journal of Pediatrics*.

[4] Clarke S. L. N., Bowron A., Gonzalez I. L. (2013). Barth syndrome. *Orphanet Journal of Rare Diseases*.

[5] Taylor C., Rao E. S., Pierre G. (2022). Clinical presentation and natural history of Barth syndrome: an overview. *Journal of Inherited Metabolic Disease*.

[6] Jefferies J. L. (2013). Barth syndrome. *American Journal of Medical Genetics Part C: Seminars in Medical Genetics*.

[7] Storch E. A., Keeley M., Merlo L. J. (2009). Psychosocial functioning in youth with Barth syndrome. *Children's Health Care*.

[8] Barth P. G., Valianpour F., Bowen V. M. (2004). X-linked cardioskeletal myopathy and neutropenia (Barth syndrome): an update. *American Journal of Medical Genetics Part A*.

[9] NORD (2019). *Barth syndrome*. https://rarediseases.org/rare-diseases/barth-syndrome/.

[10] Hornby B., McClellan R., Buckley L., Carson K., Gooding T., Vernon H. J. (2019). Functional exercise capacity, strength, balance and motion reaction time in Barth syndrome. *Orphanet Journal of Rare Diseases*.

[11] Spencer C. T., Bryant R. M., Day J. (2006). Cardiac and clinical phenotype in Barth syndrome. *Pediatrics*.

[12] Ren M., Phoon C. K. L., Schlame M. (2014). Metabolism and function of mitochondrial cardiolipin. *Progress in Lipid Research*.

[13] Schlame M., Towbin J. A., Heerdt P. M., Jehle R., DiMauro S., Blanck T. J. J. (2002). Deficiency of tetralinoleoyl-cardiolipin in Barth syndrome. *Annals of Neurology*.

[14] Lim Y. (2023). Impact of raising children with rare diseases on parental quality of life and family functioning. *International Journal of Rare Disease and Disorders*.

[15] Hays R. D., Vickrey B. G., Hermann B. P. (1995). Agreement between self reports and proxy reports of quality of life in epilepsy patients. *Quality of Life Research*.

[16] Kaplan R. M., Karoly P. (1985). Quality of life measurement. *Measurement Strategies in Health Psychology*.

[17] American Occupational Therapy Association (2020). Occupational therapy practice framework: domain and process–fourth edition. *American Journal of Occupational Therapy*.

[18] Ammann-Schnell L., Groeschel S., Kehrer C., Frölich S., Krägeloh-Mann I. (2021). The impact of severe rare chronic neurological disease in childhood on the quality of life of families-a study on MLD and PCH2. *Orphanet Journal of Rare Diseases*.

[19] Hsieh R. L., Huang H. Y., Lin M. I., Wu C. W., Lee W. C. (2009). Quality of life, health satisfaction and family impact on caregivers of children with developmental delays. *Child: Care, Health and Development*.

[20] Limbers C. A., Ripperger-Suhler J., Boutton K., Ransom D., Varni J. W. (2011). A comparative analysis of health-related quality of life and family impact between children with ADHD treated in a general pediatric clinic and a psychiatric clinic utilizing the PedsQL. *Journal of Attention Disorders*.

[21] Panepinto J. A., Hoffmann R. G., Pajewski N. M. (2009). A psychometric evaluation of the PedsQL^TM^ Family Impact Module in parents of children with sickle cell disease. *Health and Quality of Life Outcomes*.

[22] Varni J. W., Sherman S. A., Burwinkle T. M., Dickinson P. E., Dixon P. (2004). The PedsQL Family Impact Module: preliminary reliability and validity. *Health and Quality of Life Outcomes*.

[23] Mano K. E. J., Khan K. A., Ladwig R. J., Weisman S. J. (2011). The impact of pediatric chronic pain on parents' health-related quality of life and family functioning: reliability and validity of the PedsQL 4.0 Family Impact Module. *Journal of Pediatric Psychology*.

[24] Medrano G. R., Berlin K. S., Hobart Davies W. (2013). Utility of the PedsQL™ Family Impact Module: assessing the psychometric properties in a community sample. *Quality of Life Research*.

[25] Scarpelli A. C., Paiva S. M., Pordeus I. A., Varni J. W., Viegas C. M., Allison P. J. (2008). The pediatric quality of life inventory^TM^ (PedsQL^TM^) Family Impact Module: reliability and validity of the Brazilian version. *Health and Quality of Life Outcomes*.

[26] American Educational Research Association (2014). *Standards for Educational and Psychological Testing*.

[27] Cronbach L. J. (1951). Coefficient alpha and the internal structure of tests. *Psychometrika*.

[28] Taylor R. R. (2017). *Kielhofner’s Research in Occupational Therapy: Methods of Inquiry for Enhancing Practice*.

[29] Rosenbaum P. R., Rubin D. B. (1983). Assessing sensitivity to an unobserved binary covariate in an observational study with binary outcome. *Journal of the Royal Statistical Society: Series B (Methodological)*.

[30] Rosenbaum P. R., Rubin D. B. (1984). Reducing bias in observational studies using subclassification on the propensity score. *Journal of the American Statistical Association*.

[31] Abadie A., Imbens G. W. (2011). Bias-corrected matching estimators for average treatment effects. *Journal of Business & Economic Statistics*.

[32] Rahman A. A., Mohamad N., Imran M. K. (2011). A preliminary study on the reliability of the Malay version of PedsQL™ Family Impact Module among caregivers of children with disabilities in Kelantan, Malaysia. *The Malaysian Journal of Medical Sciences: MJMS*.

[33] Siegel A. F. (2016). *Practical Business Statistics*.

